# Regeneration of the heart: from molecular mechanisms to clinical therapeutics

**DOI:** 10.1186/s40779-023-00452-0

**Published:** 2023-04-26

**Authors:** Qian-Yun Guo, Jia-Qi Yang, Xun-Xun Feng, Yu-Jie Zhou

**Affiliations:** Beijing Key Laboratory of Precision Medicine of Coronary Atherosclerotic Disease, Beijing Institute of Heart Lung and Blood Vessel Disease, Clinical Center for Coronary Heart Disease, Department of Cardiology, Beijing Anzhen Hospital, Capital Medical University, Beijing, 100029 China

**Keywords:** Heart regeneration, Cardiac disease, Therapeutics, Signaling mechanisms

## Abstract

Heart injury such as myocardial infarction leads to cardiomyocyte loss, fibrotic tissue deposition, and scar formation. These changes reduce cardiac contractility, resulting in heart failure, which causes a huge public health burden. Military personnel, compared with civilians, is exposed to more stress, a risk factor for heart diseases, making cardiovascular health management and treatment innovation an important topic for military medicine. So far, medical intervention can slow down cardiovascular disease progression, but not yet induce heart regeneration. In the past decades, studies have focused on mechanisms underlying the regenerative capability of the heart and applicable approaches to reverse heart injury. Insights have emerged from studies in animal models and early clinical trials. Clinical interventions show the potential to reduce scar formation and enhance cardiomyocyte proliferation that counteracts the pathogenesis of heart disease. In this review, we discuss the signaling events controlling the regeneration of heart tissue and summarize current therapeutic approaches to promote heart regeneration after injury.

## Background

**Fig. 1:**
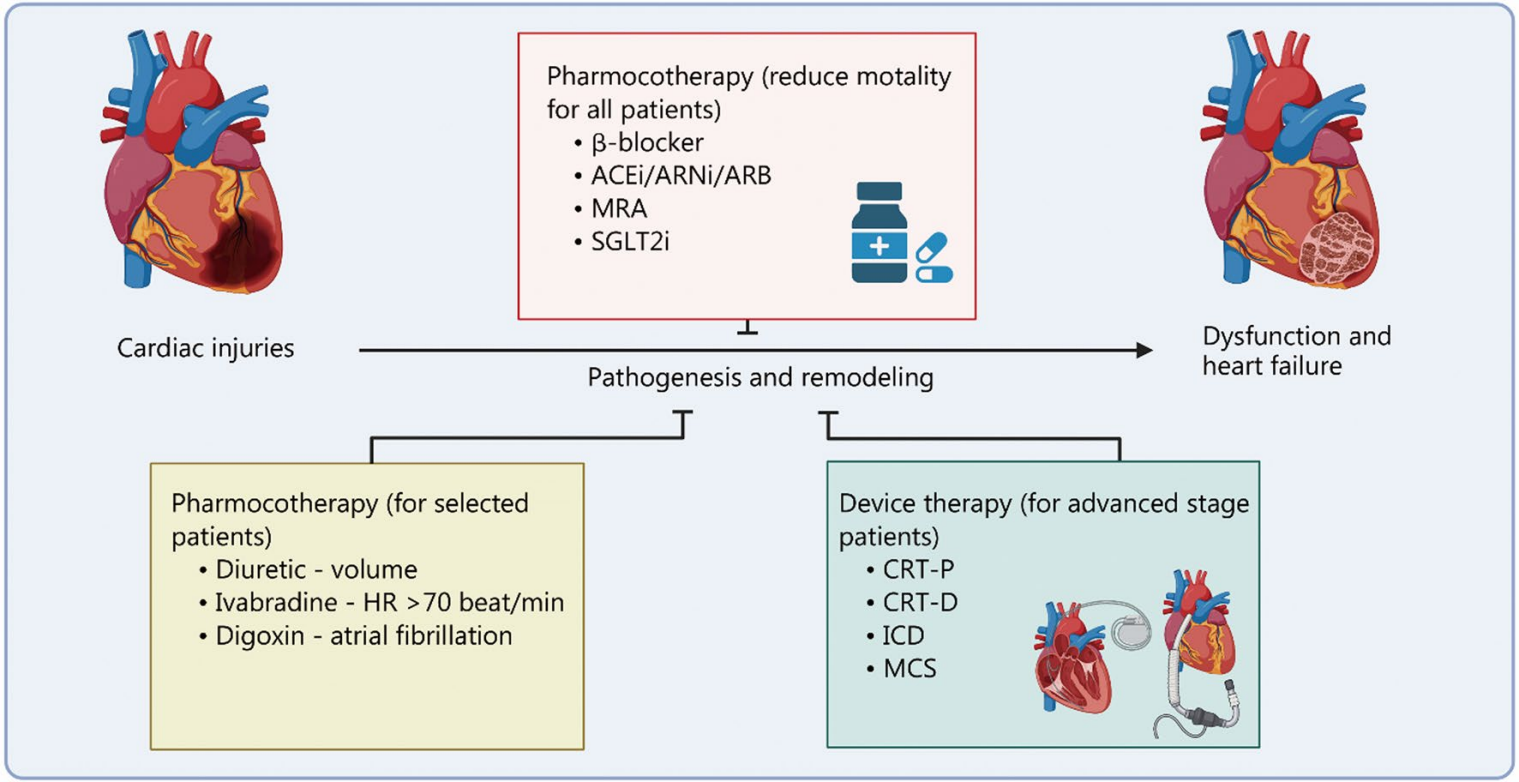
Standard of care for heart failure. Pharmacological treatment and medical devices are currently being used to manage the progression of diseases. β-blocker, ACEi, MRA, and SGLT2i are usually used for all patients with heart failure in order to reduce mortality. For selected patients, diuretic, ivabradine, and digoxin might be used. For advanced stage patients, device and surgery would be recommended. ACEi angiotensin-converting enzyme inhibitor, ARNi angiotensin receptor neprilysin inhibitor, ARB angiotensin receptor blocker, MRA aldosterone receptor antagonists, SGL2i sodium glucose cotransporter 2 inhibitor, HR heart rate, CRT-P cardiac resynchronization therapy-pacemakers, CRT-D cardiac resynchronization therapy-defibrillators, ICD implantable cardioverter defibrillator, MCS mechanical circulatory support

Cardiovascular disease is the leading cause of death and accounts for approximately 32% of global deaths, resulting in the losses of 17.9 million lives each year [1, 2]. Military personnel is significantly more likely to report higher work-related stress than civilians [3, 4], contributing to the long-term development of cardiovascular diseases and acute triggering of heart failure [5]. Cardiovascular disease represents the cause of more than 10% of military pilots’ groundings [6]. The rate of heart failure among hospitalized veterans reaches as high as 0.5% [7]. These studies highlight the importance of cardiovascular research in military medicine. Despite tremendous efforts and advances in cardiovascular research and therapies, heart failure continues to maintain high mortality and morbidity rates [1, 8]. Taking longer life expectancy, higher rates of obesity, diabetes, and modern lifestyle into consideration, epidemiologic studies predicted a 46% increase in heart failure patients by 2030 [9, 10]. Figure 1 illustrates the standard of care for managing heart failure. Currently, pharmacological treatment can slow down heart failure progression, but it still needs a breakthrough.

Potential approaches for cardiac regeneration have been tested, including strategies based on in situ cellular reprogramming and de novo tissue engineering methods. Although promising data have been accumulated, each of these approaches faces challenges. Cardiomyocytes of the adult human heart are terminally differentiated and have virtually no regenerative capacity, making it hard to reboot the proliferation of cardiomyocytes after injuries [11]. Although tissue engineering approaches have developed rapidly owing to the improvement of biomaterials and 3D printing, creating a functional heart in vitro remains a great challenge [12]. Stem cell-based therapies attempt to promote heart regeneration by injecting stem cells into patients. However, the survival, anchor, differentiation, and maturation of stem cells at the injured site are hard to control, and thus require further optimization before being ready for clinical practice [13, 14]. Recent studies suggest that the substances secreted by stem cells may promote heart regeneration [15, 16], initiating the search for drugs that target the molecular signaling pathways induced by these substances. Therefore, further understanding the molecular mechanism controlling heart regeneration will help to facilitate the emergence of new therapies that could restore cardiac function. This review summarizes the molecular signaling pathways for heart regeneration and discusses the progress and challenges of approaches for heart regeneration.

## Role of molecular signalings in heart regeneration

### Notch and Notch intracellular domain (NICD) promote cardiomyocyte proliferation and inhibit immune cell infiltration

**Fig. 2:**
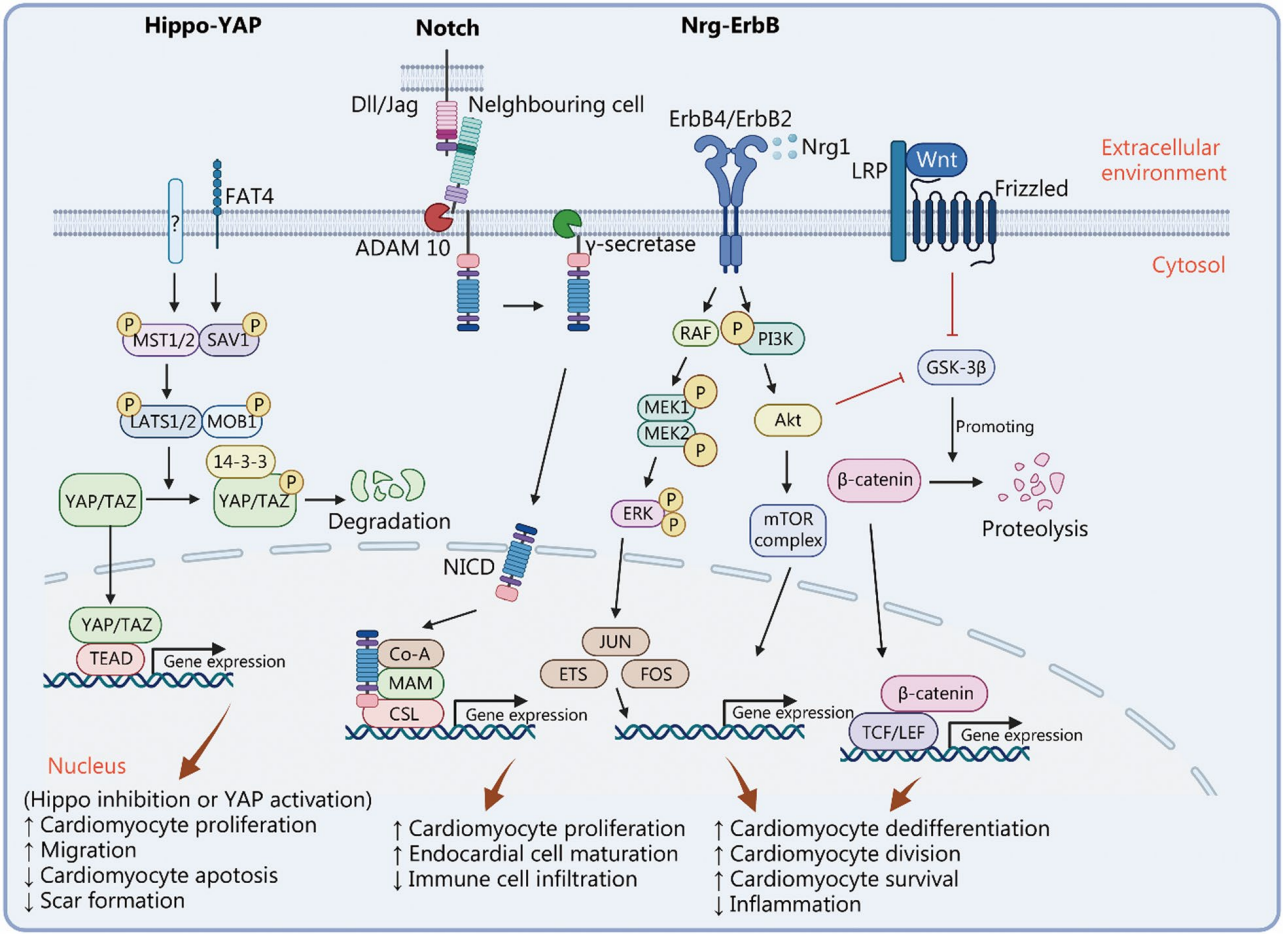
Signaling pathways in heart repair and regeneration. Hippo-YAP, Notch and Nrg-ErbB signaling pathways are the major players in regulating heart repair and regeneration after injuries. Hippo-Yap regulates cardiomyocyte proliferation, migration, and apoptosis, thus affecting scar formation after injury. Notch signaling controls cardiomyocyte proliferation, as well as immune cell infiltration and endocardial cell maturation. Nrg-ErbB signaling affects cardiomyocyte dedifferentiation, division, and survival. FAT4 FAT atypical cadherin 4, MST macrophage stimulating, SAV1 salvador family domain-containing protein 1, LATS large tumor suppressor kinase, MOB1 MOB kinase activator 1, YAP Yes-associated protein, TAZ tafazzin, phospholipid-lysophospholipid transacylase, TEAD TEA domain family, ADAM ADAM metallopeptidase domain, NICD Notch intracellular domain, MAM mastermind, CSL citrate synthase like, ErbB2 Erb-B2 receptor tyrosine kinase, RAF v-raf-leukemia viral oncogene, PI3K phosphatidylinositol 3-kinase, MEK1 mitogen-activated protein kinase kinase 1, ERK extracellular signal-regulated kinase, Akt protein kinase B, mTOR mechanistic target of rapamycin kinase, JUN Jun proto-oncogene, ETS ETS transcription factor family, FOS FBJ osteosarcoma oncogene, LRP LDL receptor related protein, GSK-3β glycogen synthase kinase-3 beta, TCF T-cell factor, LEF lymphoid enhancer factor

Heart regeneration was first described in zebrafish 20 years ago by Poss et al. [17]. Since this milestone study, the underlying signaling pathways have been extensively studied, as summarized in Fig. 2, first, showing that Notch mediates heart generation [18]. Since then, efforts have been made to understand the signaling events boosting cardiomyocyte proliferation, with the hope of aiding human heart regeneration. Notch signaling plays an important role in regulating endocardium maturation via serpine1. Inhibiting or activating Notch both result in impairment of heart regeneration, indicating a dynamic change of Notch activity is crucial [19]. In addition, Notch signaling in the endocardium interacts with cardiomyocytes as an antagonist for Wnt signaling and promotes cardiomyocyte proliferation [20]. Following the initial inflammatory response, the endocardium and epicardium regenerate first to provide the right environment for cardiomyocyte proliferation. For example, the endocardium and epicardium secrete retinoic acid, and the epicardium produces fibronectin of extracellular matrix (ECM) [21, 22]. The newly-formed heart muscle is found to populate via cardiomyocyte dedifferentiation and proliferation [23]. A study by Gemberling et al. [24] demonstrated that neuregulin 1 (Nrg1) is up-regulated after heart injury and serves as a potent inducer of cardiomyocyte proliferation. Notch signaling is also involved in this process, and a remarkable increase in Notch1b and DeltaC expression has been observed [18]. Interestingly, both Notch inhibition and Notch overexpression have been found to inhibit cardiomyocyte proliferation and heart regeneration, suggesting a delicate balance of this pathway is required [25]. Further studies by Pfefferli et al. [26] and Gupta et al. [27] have distinguished the contribution of different layers of cardiomyocytes during regeneration. Fate mapping with *careg:EGFP* has shown that the primordial cardiac layer incompletely regenerates after cryoinjury and grow restrictively by lateral expansion, while cortical and trabecular layers are primarily responsible for myocardium growth. When overexpressed specifically in cardiomyocytes, Notch also improves cardiac function by reducing the formation of scars [28]. Notch signaling pathway as a potential target for therapeutic approaches has been recently discussed [29]. Functional screening of congenital heart disease risk loci shows that *maml3* mutants can decrease cardiomyocyte proliferation through inhibition of Notch signaling [30], indicating that overexpression of *maml3* may induce cardiomyocyte proliferation by activating Notch.

### Hippo and Yes-associated protein (YAP) regulate cardiomyocyte proliferation and scar formation

The Hippo-YAP pathway is highly conserved and plays a pivotal role in cardiomyocyte cell cycle re-entry. Hippo deficiency enhances cardiomyocyte regeneration and heart functional recovery while reducing scar formation after myocardial infarction in adult mice [31, 32]. The Hippo-deficient cardiomyocytes express higher levels of proliferative and stress response genes, such as *Park2* [32]. YAP, the inactivated downstream effector of Hippo, is abundant in neonatal heart tissue but not in adult heart tissue. Recent studies found YAP to be a key regulator for cardiac development and regeneration in mice [33–35]. Similar to inhibiting Hippo, activation of YAP results in less scar formation and improved heart function after myocardial infarction at postnatal days 7 and 28 as well as adult stages [35, 36]. In Erb-B2 receptor tyrosine kinase (ErbB2)-overexpressed mice, YAP mediated a robust epithelial-mesenchymal transition (EMT)-like regeneration by interacting with the cytoskeleton and altering the mechanical characteristics of the cell [33]. In addition, non-coding RNAs make up a major part of the complex regulatory signaling network. Eulalio et al. [37] found that miR-199a and miR-590 can effectively induce cell cycle re-entry of cardiomyocytes in vitro as well as in neonatal and adult mice. In murine myocardial infarction models, overexpression of miR-199a and miR-590 via single-dose injection of synthetic RNA promotes cardiac regeneration and recovery of cardiac function [37, 38]. Recently, Gabisonia et al. [39] found that, using infarcted pig hearts, miR-199a was shown to facilitate cardiac repair and increase muscle mass and contractility. Follow-up studies on miR-199a have identified potential downstream signaling, such as CD151 [40], mechanistic target of rapamycin (mTOR) [41] and Wnt2 [42]. Cardiac-specific overexpression of miR-128 in neonatal mice attenuates cardiomyocyte proliferation and functional recovery after myocardial infarction. miR-128 regulates cardiomyocyte cell cycle re-entry via SUZ12, a chromatin modifier that targets p27, cyclin E, and CK2 [43]. Overexpression of miR-195 (a member of miR-15) leads to reduced proliferation and hypertrophy of cardiomyocytes, while inhibition of the miR-15 family increases cardiomyocyte proliferation after myocardial infarction in adult mice. The downstream target of miR-195 includes cell cycle genes, mitochondrial genes, and inflammatory genes [44]. Similarly, miR-1/-133a is also a negative regulator of cardiomyocyte cell cycle re-entry in the adult heart. Short-term deletion of miR-1/-133a protects against myocardial infarction. However, long-term deficiency leads to heart failure [45]. circNfix, a circular RNA, is up-regulated in the adult hearts of humans and mice. Knocking down circNfix releases suppression on downstream cyclinA2 and cyclinB1 and increases miR-214 activity, leading to enhanced cardiomyocyte proliferation and recovery after injury [46]. miR-152 has been found to be a target of Toll-like receptor 3 (TLR3) and induces cardiomyocyte proliferation by regulating cell cycle proteins downstream of YAP1 [47]. Recent study shows that FAM122A, an endogenous inhibitor of protein phosphatase 2A, is a novel regulator in the mesendodermal specification and cardiac differentiation via Hippo and Wnt signaling pathways [48]. In the first step, RNA-binding protein LIN28a stimulates the formation of new cardiomyocytes and prevents cardiomyocyte apoptosis [49]. Activation of YAP promotes progenitor regeneration by triggering LIN28a transcription [50].

To date, little is understood about the removal of the scar and the functional integration of regenerated cardiomyocytes. The collagenolytic activity was detected in the injured region between day 14 to 30. In the same period, expressions of matrix metalloproteins (MMPs), such as MMP-2 and MMP-14a, are up-regulated, suggesting a potential role for them in scar removal [51]. Expression of miR-101a is inhibited after the onset of injury but up-regulated between days 7 to 14. Suppression of miR-101a promotes cardiomyocyte proliferation but inhibits scar removal. Depletion of the downstream target gene *Fosab* rescued the scar-clearing defect of miR-101a inhibition, demonstrating that miR-101a regulates scar removal via *Fosab* [52]. Scar formation is regulated by YAP signaling, and macrophages directly produce collagen to make up the scar [53, 54]. Deletion of YAP from zebrafish does not affect the proliferation of cardiomyocytes but leads to larger injuries, showing that initial scar formation is important to control the damage [53]. In zebrafish, fibrosis does not preclude scar-free regeneration [55, 56].

### ErbB/PI3K/ERK and Wnt/β-catenin control cardiomyocyte proliferation, dedifferentiation, and inflammation

The Nrg1/ErbB has been recognized as a potential signaling pathway involved in the heart regeneration program. Nrg1 was initially proposed for its potential relevance to mitogenic effects in mammalian cardiomyocytes and further was proved in the post-injured zebrafish heart by Gemberling et al. [24], which provided the foundation for mouse experiments and clinical trials. In adult mice, injection of Nrg1 induces cell cycle re-entry and cardiomyocyte division. Inactivation of the tyrosine receptor ErbB4 for Nrg1 reduces cardiomyocyte proliferation, while stimulation of ErbB4 enhances it [57]. The deletion of another co-receptor for Nrg1, ErbB2, also shows its importance for cardiomyocyte proliferation in neonatal mice. Constitutive activation of ErbB2 in both neonatal and adult mice leads to cardiomyocyte proliferation and dedifferentiation via extracellular signal-regulated kinase (ERK), protein kinase B (Akt) and glycogen synthase kinase-3 beta (GSK-3β)/β-catenin downstream signaling. Notably, transient activation of ErbB2 promotes regeneration after myocardial infarction in mice [58].

The initial inflammatory response is required for complete regenerative capacity. Anti-inflammatory treatment reduces cardiomyocyte proliferation and impairs the vascularization of newly-formed tissue, resulting in an inability to clear the fibrotic deposition [59]. In contrast, the immune cell is not required for cardiomyocyte mitotic activity under normal conditions [59]. Fang et al. [60] have found that inflammatory cytokines promote cardiomyocyte proliferation via activating JAK1/STAT3 signaling. Inhibiting this signaling by expressing a dominant negative form of STAT3 leads to a reduction in cardiomyocyte proliferation. MAPK/ERK acts as a critical signaling for vertebrate tissue regeneration; its potential roles in tissue engineering and regenerative medicine have been emphasized [61]. Kynurenine stimulates cardiomyocyte proliferation by activating the cytoplasmic aryl hydrocarbon receptor-SRC-YAP/ERK pathway; it also stimulates cardiac angiogenesis by facilitating aryl hydrocarbon receptor nuclear translocation and increasing vascular endothelial growth factor A (VEGF-A) expression [62]. Dual-specificity phosphatase 6 (DUSP6), which can dephosphorylate ERK1/2, is a regenerative repressor during zebrafish heart regeneration [63]. Deletion of *Dusp6* in mice improves cardiac outcomes by reducing neutrophil-mediated myocardial damage induced by myocardial infarction-caused inflammation [64]. Furthermore, a DUSP6 inhibitor has been tested in myocardial infarction rats, showing that it improves heart function and suppresses inflammatory cardiac remodeling [65]. In addition, the cardiac-derived ECM may provide an ideal scaffold for heart tissue engineering [66], and nuclear pore numbers are decreased during cardiomyocyte maturation, and this reduces nuclear responses to activation of MAPK induced by extracellular signals [67]. Activation of Nrf1, a stress-responsive transcription factor is seen in regenerating cardiomyocytes. *Nrf1* overexpression can protect the heart from ischemic injury, while deletion inhibits neonatal heart regeneration by affecting proteasome and redox balance [28]. The role of Wnt in promoting cardiomyocyte differentiation has been further investigated, showing that it may provide a powerful tool for stem cell-based regeneration therapy [68]. These studies suggest that the molecular events initiated by extracellular signals may have therapeutic benefits for heart regeneration.

## Approaches and challenges for heart regeneration

The fate mapping experiments in mice have shown that new cardiomyocytes originate from pre-existing ones, during homeostasis [69], after injury in adults [69, 70], and during neonatal heart regeneration [71]. In addition, using a transgenic line of hypoxia-inducible factor-1α (HIF-1α), Kimura et al. [70] showed that hypoxic cardiomyocytes exhibit characteristics of neonatal heart cells and contribute mostly to cardiomyocyte formation in adults. Despite these results, many efforts have been focused on the c-Kit^+^ progenitor cells from the bone marrow [72], which were later shown to play a negligible role in heart regeneration [73]. Using the Cre/lox system and a reporter line, endogenous c-Kit^+^ cells are found to generate cardiomyocytes at a percentage less than 0.03. Although c-Kit^+^ cells contribute to the revascularization of cardiac endothelial cells, their role in myocardium regeneration is insignificant.

In order to develop new therapies, recent studies have worked on understanding the regulatory role of non-muscle cells, such as immune cells, endothelial cells, and cardiac fibroblasts. In neonatal mice, CD4^+^ regulatory T cells (Tregs) are necessary for cardiac regeneration. Depletion of Tregs inhibits cardiomyocyte proliferation and induces fibrosis, whereas adoptive transfer of Tregs rescues this phenotype [74]. Interestingly, ablation of CD4^+^ Tregs in mice at postnatal day 8 promotes heart regeneration after resection [75], suggesting the role of immune cells might differ by stages. Endothelial cells support heart regeneration by reassembling arteries, which serve as a scaffold for cardiomyocyte repopulation and also reperfuse the ischemic tissues [76, 77]. Endothelial cell migration is induced by the CXCL12-CXCR4 signaling pathway. Genetic inhibition of this signaling leads to formation of larger scars and the reduction of cardiomyocyte proliferation after myocardial infarction [76]. Consistent with this, inhibition of revascularization in zebrafish with dominant negative VEGF-A also hindered regeneration, suggesting that endothelial cells are actively engaged in cardiomyocyte proliferation [78]. Cardiac fibroblasts deposit ECM and their number increases during development and diseases, such as heart failure [79]. Transcriptomic analysis showed different gene expression profiles between fetal and adult fibroblasts of humans, suggesting fibroblasts might be potential contributors to embryonic heart regeneration [80]. However, ablating activated fibroblasts in mice has a protective effect after acute injuries [81], which contradicts its vital function in promoting heart regeneration of zebrafish [82]. This could potentially be explained by the existence of different sub-clusters of fibroblasts in the heart, but further studies are still needed [83]. In summary, modulating immune cells, endothelial cells, and fibroblasts after injury may promote cardiac regeneration and lead to further mitigation of disease.

The regenerative ability of the mammalian heart is lost during development. In humans, the scar-free repair of the heart is feasible, but only at early developmental stages [84, 85]. A case report of a newborn child by Haubner et al. [86] showed strong regeneration ability after severe myocardial infarction and tissue damage. The cardiac function of this 1-year-old child recovered a few weeks after the initial injury. Similar responses have been seen in other cases by Cesna et al. [87], Deutsch et al. [88], and Farooqi et al. [89], leading to the hope of repairing a damaged adult heart by reactivating regenerative processes that are present during the neonatal stage. Similar to humans, mice lose the capacity for heart regeneration during the early postnatal stage between postnatal days 1 to 7 [84]. A well-designed study by Drenckhahn et al. [90] showed that embryonic cardiomyocytes are able to re-enter the cell cycle and proliferate to form heart muscles. In this study, the X-linked gene *Hccs* was deleted specifically in the heart muscle; this deletion is lethal for the cell. In heterozygous females (half of the cardiomyocytes were normal due to random X inactivation), the mutant cells contributed to less than 10% of tissue volume, showing that the normal cardiomyocytes are able to regenerate about 50% loss of cardiomyocytes at embryonic stage [90]. By removing 10% of the ventricle from mice at various ages, the time windows of regeneration are characterized [71]. The murine heart can regenerate at postnatal day 1 after surgical resection with minimal scar or hypertrophy [91]. This regenerative ability is continuously lost until it ceases at postnatal day 7. In support of this conclusion, similar results have been observed in many other injury models by Haubner et al. [92] and Porrello et al. [44] although the collagen scar has been observed when resecting a larger part (20%) of the ventricle [93]. A study by Porrello et al. [44] using left anterior descending artery (LAD) ligation-induced injury showed that the heart regenerates within 3 weeks after extensive necrosis. This study compared changes in gene expression after injury between postnatal days 1, 3, and 10. Many genes regulating mitosis, cell division, cell cycle, and ECM synthesis have been identified, including *NPPA* (atrial natriuretic factor), *Nanog* (stem cell marker), and *HIF3A* (hypoxia-inducible factor-3α gene) [92]. Further study by Darehzereshki et al. [91] with cryoinjury models has revealed different modes of repair after different types of injury. Neonatal hearts are able to regenerate after non-transmural cryoinjury but not after transmural injury and differential plasminogen activator inhibitor 1 (PAI-1) expression could be a potential explanation. Konfino et al. [94] found that both neonatal and adult mice respond differently to LAD-induced myocardial infarction and resection. The adult heart forms a thin scar after myocardial infarction, whereas apical resection leads to the occurrence of a hemorrhagic scar. Together, these findings suggest that different treatments should be developed to administer to specific injuries.

The limitation of this model is the lack of cell death, inflammation, and debris clearance steps during the healing process [95]. Cryoinjury is one of the most commonly used methods, in which a precooled metal is used to freeze part of the ventricle [55, 56]. Although cardiac tissue loss is similar to the resection model, it takes much longer, around 130 d, to regenerate the heart after cryoinjury [56]. Genetic models of cardiomyocyte death have also been used to study heart regeneration in zebrafish. Wang et al. [96] ablated cardiomyocyte with the expression of cytotoxic diphtheria toxin A chain, induced by cell-specific cyclization recombination enzyme (Cre). This method induces around 60% loss of cardiomyocytes while leaving the endocardium and epicardium intact, which resembles cardiomyopathy in human patients [97]. Heart function and tissue are restored in around 30 d, which could be attributed to the importance of epicardium in heart regeneration [98].

Using these injury models, the cellular processes of heart regeneration have been better characterized and a signaling network of genes was identified to be crucial for scar-free regeneration. The regenerative process can be separated into four major stages: 1) the acute reaction to injury, including recruitment of immune cells and deposition of fibrotic tissues; 2) the endocardium and epicardium regenerate in order to support the myocardium; 3) the myocardium is regenerated via proliferation, and 4) the functional integration of newly generated cardiomyocytes, scar removal, and inflammation resolution [95].

### Transplantation of progenitor-derived cells and stem cells

Cell transplantation to repair the injured heart has been started for more than a decade. Intracoronary administration of bone marrow-derived progenitor cells can improve the recovery of left ventricular contractile function in patients with acute myocardial infarction [99]. However, studies with double-blind randomized designs show that injection of bone marrow mononuclear cells fails to improve the left ventricular contractile function [100–102]. The randomized placebo-controlled study of myoblast transplantation also shows that myoblast injections are unable to improve echocardiographic heart function [103]. Adverse effects such as arrhythmias are always problematic, as skeletal myoblasts are not able to conduct electromechanical signals as cardiomyocytes [104]. Therefore, efficient treatment may be cell-specific and achieved by transplantation of progenitor-derived cells. Recent studies have graded mesoderm assembly controls cell fate and morphogenesis of the early mammalian heart [105].

Another approach is to induce the differentiation of cardiomyocytes in vitro using embryonic stem cells (ESCs) or induced pluripotent stem cells (iPSCs). Both cell types are able to succeed in vitro to produce cardiomyocyte-like cells [106, 107]. Convincing evidence shows that transplantation of ESC-derived cardiomyocytes improves heart function by integrating with pre-existing cardiomyocytes to transduce electromechanical signals [108, 109]. Although transplantation of human ESC-derived cardiomyocytes can regenerate the infarcted pig heart, it induces ventricular tachyarrhythmias [110]. There have been few clinical trials in humans given the ethical challenges of ESCs as well as concerns about side effects. One trial shows some positive results, but with an overall low engraft rate and lack of careful characterization of the control group [111]. Similarly, another trial shows that transplantation of iPSC-derived cardiomyocytes improves ventricular contractility and promotes heart regeneration, but has low engraftment and survival rate of cardiomyocytes, and induces complications such as tachycardia [112, 113].

The POSEIDON study shows that bone marrow-derived mesenchymal stem cells (MSCs) may have cardiogenic potential and improve the functional capacity of the heart [114]. However, the conclusion is hindered by the lack of a placebo control group and a small patient cohort of 30. However, a randomized double-blind trial shows that bone marrow-derived mesenchymal stromal cells produce a moderate improvement in left ventricular ejection fraction (LVEF) and stroke volume of ischemic heart [115]. Similar results have been reported in trials using MSCs derived from different sources, such as the umbilical cord-mesenchymal stem cell (UC-MSC) [116]. The Congestive Heart Failure Cardiopoietic Regenerative Therapy (CHART-1) trial demonstrated that MSC injection is overall safe [117] and has long-term benefits in patients with significant left ventricular enlargement [118, 119]. The recent CONCERT-HF trial shows that MSC in combination with c-Kit positive cells (CPCs) can significantly reduce heart failure-related major adverse cardiac events (HF-MACE). However, no improvement in left ventricular function or reduction of scar size can be achieved, requiring further elucidation of the underlying mechanism [120, 121]. Other clinical trials show that MSC injection fails to produce functional improvement of the heart [122, 123]. Although MSCs can differentiate into cardiomyocytes in vitro [124], MSC-derived endothelial cells are the main contributor to heart regeneration in animal model [125]. A randomized double-blind multi-center trial TEAM-AMI shows that the efficacy of MSC injection is highly dependent on the microenvironment [126], supporting that the clinical benefits are mainly mediated by indirect effects instead of by generating new cardiomyocytes [123]. Vagnozzi et al. [127] showed that intracardiac injection of killed stem cells or use of chemical inducers for immune response produced similar results as live adult stem cell. All these treatments induce a regional accumulation of CCR2^+^ and CX3CR1^+^ macrophages, which affect fibroblasts and the ECM at the injury site. A series of animal studies by Bolli et al. [128] demonstrated that transplanted cells cannot engraft into the myocardium nor differentiate to cardiomyocytes, although improved cardiac function was observed. This dissociation of therapeutic improvement with engrafting rate has been seen among MSCs, ESCs, and CPCs treatment, independent of delivery method and preconditions [129]. These new findings suggest that the benefits from stem cell injection are mainly due to secreted factors instead of cell replenishment. Therefore, understanding the molecular signaling induced by factors secreted by stem cells becomes more important for treatment of heart injury. Recent studies show that endoderm-derived islet1-expressing cells can differentiate into endothelial cells to function as hematopoietic stem and progenitor cells [130], which may serve as an alternative approach for stem cell transplant; in addition, human- or animal-derived decellularized heart patches have been used in vivo and in vitro studies to promote the regeneration of heart tissue [131]. However, due to the complexity of cardiac tissue engineering, significant hard work must be done before the approaches can be clinically used.

Currently, a growing number of clinical trials [130] (see Bolli et al. [129] for a comprehensive list of trials) and Meta-analyses [132] have greatly expanded the knowledge and potential choices of cell sources and interventions for heart disease, such as IMMNC-HF with bone marrow mononuclear cell [133]; LAPiS (NCT04945018), HEAL-CHF (NCT03763136) and NCT05223894 with human iPSC derived cardiomyocytes; NCT05147766 with UC-MSCs; NCT03797092 with adipose-derived stromal cell; and BioVAT-HF (NCT04396899) with engineered human myocardium. DREAM-HF, a phase III clinical trial, recruited 565 patients and upon completion will provide evidence in analyzing the efficiency of MSC injection as a heart failure treatment [14, 134, 135]. Recent studies show that human mesenchymal stromal cells and endothelial colony-forming cells reduce cardiomyocyte apoptosis, scar size, and adverse cardiac remodeling, compared to vehicle, in a pre-clinical model of acute myocardial infarction [136]. Human ESC-derived endothelial cells also attenuate cardiac remodeling in a mouse myocardial infarction model [137]. Besides cardiomyocytes, cardiac interstitial cells also play crucial roles during cardiac regeneration [138], which opens another avenue to improve heart regeneration. These studies provide useful information for cell therapy approach to treat cardiac injury in the future.

### Inducing proliferation of existing cardiomyocytes

The safest and least immunogenic option for cardiac regeneration is using pre-existing cardiomyocytes, although human cardiomyocytes are well-known for being non-proliferative [85]. There is evidence supporting that cardiomyocytes self-renew at a slow but steady speed [69], and previous mechanistic studies in mice and zebrafish have provided clues for potential therapeutic targets. Combined expression of cell cycle-related genes, *Cdk1*, *Ccnb*, *Cdk4,* and *Ccnd* induces post-mitotic cell proliferation and improves ventricular function after myocardial infarction [139]. As discussed earlier, the Hippo-YAP pathway is a promising target for promoting cardiomyocyte proliferation. Adeno-associated virus (AAV)-based genetic knockdown of Hippo pathway gene *Sav* in pig models has been shown to increase the renewal rate of cardiomyocytes after myocardial infarction and improve LVEF [140]. No arrythmia, tumor formation, or mortality has occurred after treatment, making this a promising approach to advancing clinical trials.

Another potential target is Myc, a transcription factor involved in cell replication, differentiation, metabolism, and apoptosis [141]. Four-hour acute activation of Myc signaling in juvenile mice leads to a marked proliferative response in vivo [142]. Mechanistically, this effect is mediated by positive transcription elongation factor b (P-TEFb), which consists of CDK9 and cyclinT1. Furthermore, a transient cardiomyocyte-specific expression of Myc, SRY-box transcription factor 2 (SOX2), OCT4 (named POU5F1; POU domain, class 5, transcription factor 1), and KLF transcription factor 4 (KLF4) can induce dedifferentiation of adult cardiomyocytes characterized by a gene expression profile resembling that of fetal cells. This allows the reprogrammed cardiomyocytes to re-enter the cell cycle and divide into more cardiomyocytes, leading to improved cardiac function after myocardial infarction [143]. Prolonged expression of these four factors resulted in tumor formation and lethality in mice, however, urging the need for more in-depth studies to avoid potential safety issues.

The Nrg1 has shown its mitogenic effect in pre-existing cardiomyocytes (mentioned in section “ErbB/PI3K/ERK and Wnt/β-catenin control cardiomyocyte proliferation, dedifferentiation, and inflammation”). Furthermore, Polizzotti et al. [144] show that recombinant neuregulin 1 (rNRG1) induces the proliferation of cardiomyocytes both in mice and in isolated human myocardium, which opened the therapeutic window and prompted clinical trials. A double-blind, placebo-controlled clinical trial of neuregulin 1β3 (cimaglermin alfa) shows sustained improvements in LVEF [145]. Another clinical trial shows that recombinant human neuregulin 1 (rhNRG1) can increase LVEF and decrease end-diastolic volume (EDV) and end-systolic volume (ESV) in chronic heart failure patients. However, these results were statistically indistinguishable from the placebo, and it remains unclear if this treatment improves heart function by inducing regeneration [146]. Overall, there is active research underway to develop and optimize therapies using identified gene targets and to explore new targets, i.e.*,* Hoxb13 by Nguyen et al. [147], Meis1 by Mahmoud et al. [148], and miR-199a by Eulalio et al. [37] and Gabisonia et al. [39].

### Reprogramming non-muscle cells into cardiomyocytes

Reprogramming other cells of the heart, such as fibroblasts, into cardiomyocytes, is another way to achieve the challenging task of repairing the heart. As a large cell population of the heart [149], fibroblasts are the first responders after cardiac injuries, thus making them an ideal source of cardiomyocytes. Forced expression of cardiac transcription factor combinations, such as GATA binding protein 4 (GATA4), myocyte enhancer factor 2C (MEF2C), and T-box transcription factor 5 (TBX5) (GMT cocktail) [150]; or GATA4, heart and neural crest derivatives expressed transcript 2 (HAND2), MEF2C and TBX5 (GHMT) [151], can successfully transform fibroblasts into cardiomyocytes in vitro. Bypassing the iPSC stage, this approach reprograms fibroblasts directly into contractile cardiomyocytes that express typical cardiomyocyte markers. In vivo expression of GHMT using retroviral infection in mice showed that reprogramed cells can form cardiomyocytes and conduct electromechanical signals after myocardial infarction induced by LAD ligation [151]. Many genes and signaling pathways involved in heart regeneration also modulate reprogramming of fibroblast into cardiomyocytes, including Notch signaling [152], zinc finger transcription factor 281 (ZNF281; regulating inflammation) [153], fibroblast growth factor (FGF) and VEGF [154], Akt1/protein kinase B [155], Bmi1 (epigenetic factor) [156], and chemical factors [157]. Recently, Wang et al. [158] found that autophagic factor Beclin1 negatively regulates fibroblast reprogramming in an autophagy-independent manner, and that Beclin1 haploinsufficiency in mice promotes reprogramming and reduces scar size after myocardial infarction. In addition, a combination of miRNAs, miR-1, -133, -208, and -499 have also been found to induce cardiomyocytes from fibroblasts both in vitro and in vivo [159, 160], providing alternative targets for fibroblast reprogramming. Alternatively, Lalit et al. [161] showed that mesoderm posterior bHLH transcription factor 1 (MESP1), GATA4, TBX5, NK2 homeobox 5 (NKX2-5), and BAF60C (SMARCD3, SWI/SNF related, matrix associated, actin dependent regulator of chromatin, subfamily D, member 3) expressed in fibroblasts produce a progenitor population that gives rise to cardiomyocytes, endothelial cells, and mural cells in myocardial infarction mice models. Recent study also suggests that the cardiac gene *TBX20* (T-box transcription factor 20) enhances myocardial reprogramming induced by the MGT133 reprogramming cocktail (MEF2C, GATA4, TBX5, and miR-133) [162]. In summary, transcription factor combinations play an important role in transforming fibroblasts into cardiomyocytes in mice.

Despite the success in mice, human fibroblasts are more resistant to both the transcription factor and miRNA combination-induced reprogramming and have shown overall inadequate efficacy to produce cardiomyocytes. Furthermore, the induced cardiomyocytes mostly lack contractility [163, 164]. Follow-up studies discovered that the reprogramming process of human fibroblasts requires the addition of other factors, such as MESP1 and myocardin (MYOCD) [165, 166], ZFPM2 (zinc finger protein, FOG family member 2) [166], V-Ets erythroblastosis virus E26 oncogene homolog 2 (ETS2) and MESP1 [167]. More efforts are still needed to understand the molecular mechanism and the heterogeneity [168] of induced cardiomyocytes and improve the efficacy of this approach before clinical application. Nevertheless, studies using mouse models have reached a new level by using a novel Tcf21iCre/reporter/MGTH2A transgenic mouse system showing that cardiac reprogramming can repair myocardial infarction [169]. However, whether it is safe and efficacy for patients remains to be validated.

### Non-cell-based approaches

Although still in the early stages, approaches that are not based on cells have the great potential as they bypass the difficulties related to low engraft rates, unclear mechanism, and ethical and safety problems. Study by Puente et al. [170] in postnatal mice found that oxidative stress induces cell cycle arrest, thus contributing to the loss of heart regenerative ability. Based on this finding, Nakada et al. [171] designed experiments where mice were exposed to hypoxia for a week after myocardial infarction. This treatment triggers a robust regenerative response and improves left ventricular systolic response. Fate-mapping showed that pre-existing cardiomyocytes proliferate to form myocardium, making it an intriguing idea to treat patients with gradual systemic hypoxia.

Secreted factors, such as growth factors VEGF-A, FGF-2, Nrg1, and thymosin b4, protect against myocardial injuries in animal models [172, 173]. However, this effect has not been seen in clinical trials with both VEGF-A and FGF-2 [174, 175]. One explanation for this might be that the delivery method cannot ensure a high bioavailability, as a better recovery is achieved by using synthetic mRNA to express VEGF-A in mice [176]. Recent studies show that VEGF-A-induced angiogenic sprouting can be attenuated by siRNA knockdown or CRISPR/Cas9 knockout of LINC00607 [177]. VEGF mRNA has been administrated to patients via direct intramyocardial injection, showing that it may be safe for introducing genetic material to the cardiac muscle [178]. Nrg1 sustains the epicardial-mediated cardiac regeneration capacity of neonatal heart explants [179]. Oxytocin also activates epicardial cells and promotes heart regeneration after cardiac injury [180]. Daily administration of thymosin β4, a peptide known to restore vascularization of the epicardium [181], gives mice the capability of producing new cardiomyocytes and improves recovery after myocardial infarction [182, 183]. These studies have been confirmed by a recent report showing that thymosin β4 and also prothymosin α promote cardiac regeneration in mice [184]. Exosomes are small extracellular vesicles containing different cargoes like protein, RNA and lipids [185]. Exosomes secreted by iPSC or cardiac progenitor populations promote cardiac functional recovery in animal models [186, 187]. Furthermore, mechanistic studies by Cai et al. [188] and Zhou et al. [189] showed that the epicardium, similar to stromal stem cell, plays an important role in heart regeneration by both serving as a source for cardiomyocytes, and most importantly, by providing the required paracrine factors [190]. A proteomic study by Arrell et al. [191] comparing chronic infarction models with and without human stem cell treatment identified 283 and 450 altered proteins, respectively. This finding could provide a roadmap to future therapeutics using secreted factors. Owing to the advancement of the biomedical engineering field, new methods are being developed to efficiently deliver these factors, including exosomes [192], cardiac patches [193], and bioactive hydrogel [194]. For example, a recent report shows that cardiac tissue regeneration can be induced by the delivery of miR-126 and miR-146a via exosomes [195]. Recent studies show that cardiogel-loaded chitosan patches or injectable hydrogels containing anti-apoptotic, anti-inflammatory, and pro-angiogenic agents may have therapeutic benefits for heart injury [196, 197]. Together, the precise delivery of factors promoting myocardial proliferation and inhibiting apoptosis and inflammation has the potential to enable heart regeneration in situ.

Together, these findings provide exciting new directions for regenerative therapeutics for human heart disease. Notably, there are several barriers that need to be removed before translating these findings to clinical practice, such as the variability between species and the insufficient reproduction of results [198]. By using quantitative measurement, human-animal chimeras [199], large-animal models and platforms, i.e., CIBERCV Cardioprotection Large Animal Platform (CIBER-CLAP) [198, 200], standardized protocols and quality-control infrastructure [201], future preclinical studies are anticipated to yield positive clinical results.

## Conclusions and perspectives

**Fig. 3:**
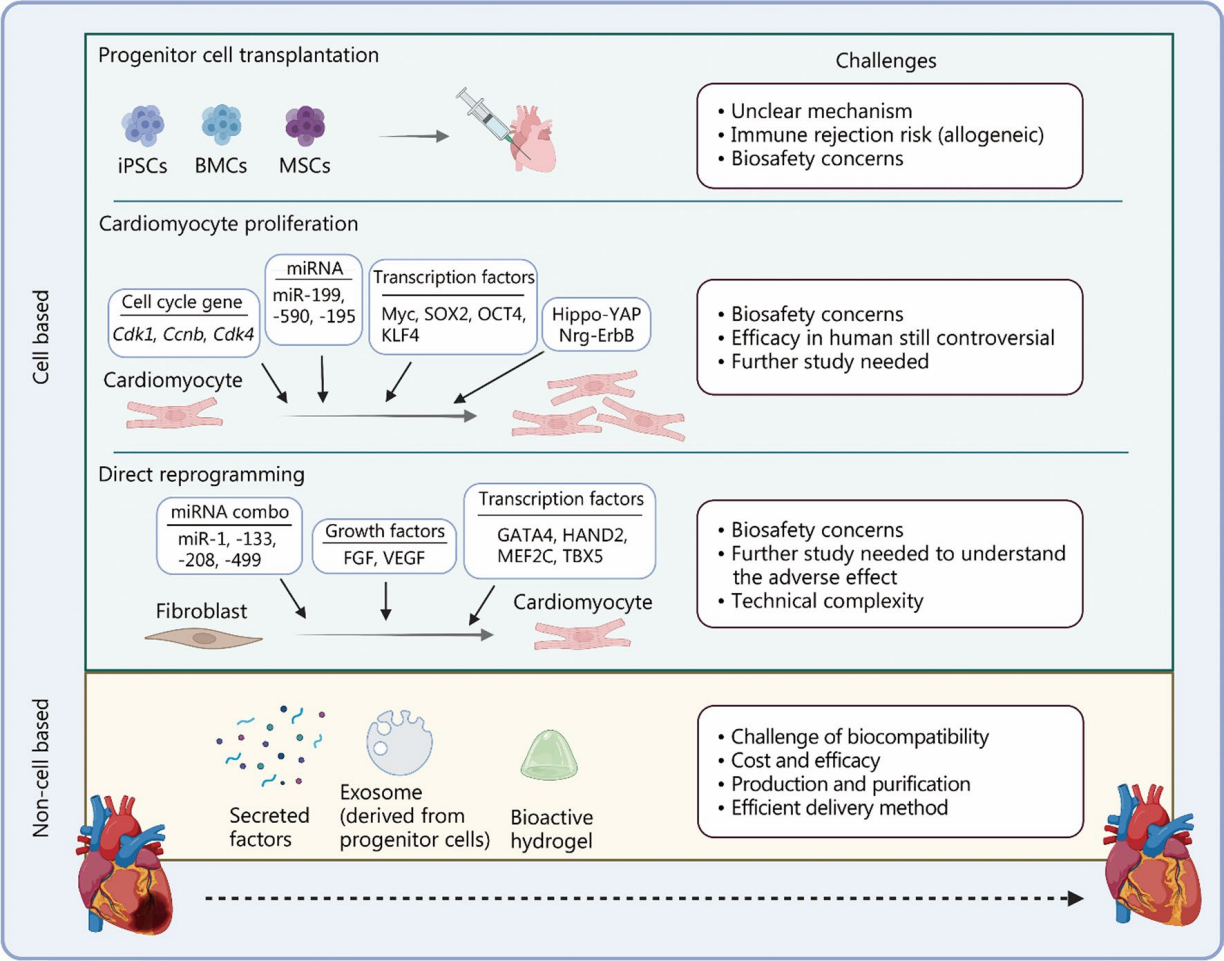
Current approaches for heart regeneration. Current attempts at heart regenerative therapies include cell based and non-cell based approaches. Each of these approaches has its own advantages and faces different challenges. iPSC induced pluripotent stem cell, BMC bone marrow cell, MSC mesenchymal stem cell, Cdk1 cyclin-dependent kinase 1, Ccnb cyclin B, SOX2 SRY-box transcription factor 2, OCT4 POU domain, class 5, transcription factor 1, KLF4 KLF transcription factor 4, YAP Yes-associated protein, Nrg neuregulin, FGF fibroblast growth factor, VEGF vascular endothelial growth factor, GATA4 GATA binding protein 4, HAND2 heart and neural crest derivatives expressed transcript 2, MEF2C Myocyte enhancer factor 2C, TBX5 T-box transcription factor 5

In summary, active research in the field has revealed common molecular mechanisms for heart regeneration and potential new targets for therapies. These potential gene targets function to regulate immune response, cardiac fibroblast activation, epicardium recovery, and cardiomyocyte proliferation after injuries. Inspired by these findings, current trials focus on inducing heart regenerative ability by cell-based approaches, including progenitor cell transplantation, inducing cardiomyocyte proliferation, and direct reprogramming. Other ongoing therapeutic explorations involve non-cell-based approaches, such as secreted factors and exosomes. In addition, the contribution of non-cardiomyocytes, such as endothelial cells and the epicardium has been actively studied. Figure 3 illustrates current approaches for heart regeneration. With studies for genetics and genomics developed gradually, gene editing technology, especially CRISPR/Cas9, has made continuous breakthroughs, which opens up a new way to manipulate the genome in vitro and in vivo, and also provides an unprecedented opportunity to explore the application of gene editing in cardiovascular diseases [202, 203]. iPSCs are increasingly being used as substitutes or supplements for animal models of cardiovascular disease [204]. Jiang et al. [205] have found that fibroblasts could be reprogrammed into cardiovascular progenitor cells using transgenic methods, which are called CRISPR-induced cardiovascular progenitor cells (ciCPCs). The implanted ciCPCs differentiate into cardiovascular cells in vivo, which significantly improve myocardial systolic function and the formation of scars, and provide a new source of cells for myocardial regeneration. With the development of artificial intelligence, Theodoris et al. [206] recently developed a machine learning approach to identify small molecules, which correct gene networks dysregulated in iPSC broadly. This approach could prevent and treat specific cardiovascular diseases in a mouse model. This study points to human–machine learning, network analysis, and iPSC technology to make this strategy feasible and potentially represent an effective path for drug discovery [206]. In addition, Lin et al. [207] demonstrated that multiplexed CRISPRi screening combined with machine learning confers functional robustness to gene expression. The prediction of synergistic enhancers by machine learning provides an effective strategy for identifying pairs of noncoding variants associated with disease-causing genes beyond the analysis of genome-wide association studies [207]. There’s a reasonable prospect that gene editing and artificial intelligence will also bring breakthroughs in heart regeneration in the future. These attempts generated promising results and could be further optimized and then tested in larger populations. Cre recombinase microinjection will help researchers identify the cell progenitors and gene networks involved in organ development [208]. A variety of tissues and organs including hearts have been produced via 3D bio-printing, which serves as in vitro models for pharmacokinetics and drug screening [209]. Although it is not promised, 3D bio-printing may be used for repairing, or even replacing, an injured heart in the future. We believe that the endeavors in fighting against heart injury will finally lead to a breakthrough for adult heart regeneration.

## Data Availability

Not applicable.

## Abbreviations

AAV: Adeno-associated virus
Akt: Protein kinase B
BAF60C: SMARCD3, SWI/SNF related, matrix associated, actin dependent regulator of chromatin, subfamily d, member 3
BMCs: Bone marrow cells
CHART-1: Congestive Heart Failure Cardiopoietic Regenerative Therapy
CIBER-CLAP: CIBERCV Cardioprotection Large Animal Platform
ciCPCs: CRISPR-induced cardiovascular progenitor cells
CPCs: C-Kit positive cells
ECM: Extracellular matrix
EDV: End-diastolic volume
EMT: Epithelial-mesenchymal transition
ErbB2: Erb-B2 receptor tyrosine kinase
ERK: Extracellular signal-regulated kinase
ESCs: Embryonic stem cells
ESV: End-systolic volume
FGF: Fibroblast growth factor
GATA4: GATA binding protein 4
GSK-3β: Glycogen synthase kinase-3 beta
HAND2: Heart and neural crest derivatives expressed transcript 2
HF-MACE: Heart failure-related major adverse cardiac events
HIF3A: Hypoxia-inducible factor 3α gene
HIF-1α: Hypoxia-inducible factor-1α
iPSCs: Induced pluripotent stem cells
KLF4: KLF transcription factor 4
LAD: Left anterior descending artery
LVEF: Left ventricular ejection fraction
MEF2C: Myocyte enhancer factor 2C
MESP1: Mesoderm posterior bHLH transcription factor 1
MSC: Mesenchymal stem cell
MYOCD: Myocardin
mTOR: Mechanistic target of rapamycin kinase
NICD: Notch intracellular domain
NKX2-5: NK2 homeobox 5
Nrg1: Neuregulin 1
OCT4: POU domain, class 5, transcription factor 1
P-TEFb: Positive transcription elongation factor b
rhNRG1: Recombinant human neuregulin 1
rNrg1: Recombinant neuregulin 1
SOX2: SRY-box transcription factor 2
TBX5: T-box transcription factor 5
Tregs: Regulatory T cells
TLR3: Toll-like receptor 3
UC-MSC: Umbilical cord-mesenchymal stem cell
VEGF-A: Vascular endothelial growth factor A
YAP: Yes-associated protein
ZFPM2: Zinc finger protein, FOG family member 2
ZNF281: Zinc finger transcription factor 281

## References

[1] Zhang Y, Lin C, Liu M, Zhang W, Xun X, Wu J (2023). Burden and trend of cardiovascular diseases among people under 20 years in China, Western Pacific region, and the world: an analysis of the global burden of disease study in 2019. Front Cardiovasc Med.

[2] Virani SS, Alonso A, Aparicio HJ, Benjamin EJ, Bittencourt MS, Callaway CW (2021). Heart disease and stroke statistics-2021 update: a report from the American Heart Association. Circulation.

[3] Pflanz S, Sonnek S (2002). Work stress in the military: prevalence, causes, and relationship to emotional health. Mil Med.

[4] Bustamante-Sánchez Á, Tornero-Aguilera JF, Fernández-Elías VE, Hormeño-Holgado AJ, Dalamitros AA, Clemente-Suárez VJ (2020). Effect of stress on autonomic and cardiovascular systems in military population: a systematic review. Cardiol Res Pract.

[5] Steptoe A, Kivimäki M (2012). Stress and cardiovascular disease. Nat Rev Cardiol.

[6] Grósz A, Tóth E, Péter I (2007). A 10-year follow-up of ischemic heart disease risk factors in military pilots. Mil Med.

[7] Heidenreich PA, Sahay A, Kapoor JR, Pham MX, Massie B (2010). Divergent trends in survival and readmission following a hospitalization for heart failure in the Veterans Affairs health care system 2002 to 2006. J Am Coll Cardiol.

[8] Mamas MA, Sperrin M, Watson MC, Coutts A, Wilde K, Burton C (2017). Do patients have worse outcomes in heart failure than in cancer? A primary care-based cohort study with 10-year follow-up in Scotland. Eur J Heart Fail.

[9] Benjamin EJ, Blaha MJ, Chiuve SE, Cushman M, Das SR, Deo R (2017). Heart disease and stroke statistics-2017 update: a report from the American Heart Association. Circulation.

[10] Lawson CA, Zaccardi F, Squire I, Ling S, Davies MJ, Lam CSP (2019). 20-year trends in cause-specific heart failure outcomes by sex, socioeconomic status, and place of diagnosis: a population-based study. Lancet Public Health.

[11] Bergmann O, Bhardwaj RD, Bernard S, Zdunek S, Barnabé-Heider F, Walsh S (2009). Evidence for cardiomyocyte renewal in humans. Science.

[12] Tenreiro MF, Louro AF, Alves PM, Serra M (2021). Next generation of heart regenerative therapies: progress and promise of cardiac tissue engineering. NPJ Regen Med.

[13] Curfman G (2019). Stem cell therapy for heart failure: an unfulfilled promise?. JAMA.

[14] Zhang J, Bolli R, Garry DJ, Marbán E, Menasché P, Zimmermann WH (2021). Basic and translational research in cardiac repair and regeneration: JACC state-of-the-art review. J Am Coll Cardiol.

[15] Plackett B (2021). Cells or drugs? The race to regenerate the heart. Nature.

[16] Tehzeeb J, Manzoor A, Ahmed MM (2019). Is stem cell therapy an answer to heart failure: a literature search. Cureus.

[17] Poss KD, Wilson LG, Keating MT (2002). Heart regeneration in zebrafish. Science.

[18] Raya A, Koth CM, Büscher D, Kawakami Y, Itoh T, Raya RM*,* et al*.* Activation of Notch signaling pathway precedes heart regeneration in zebrafish. Proc Natl Acad Sci USA. 2003;100 Suppl 1(Suppl 1):11889–95. 10.1073/pnas.1834204100 PMC304103 12909711

[19] Münch J, Grivas D, González-Rajal Á, Torregrosa-Carrión R, de la Pompa JL (2017). Notch signalling restricts inflammation and serpine1 expression in the dynamic endocardium of the regenerating zebrafish heart. Development.

[20] Zhao L, Ben-Yair R, Burns CE, Burns CG (2019). Endocardial Notch signaling promotes cardiomyocyte proliferation in the regenerating zebrafish heart through Wnt pathway antagonism. Cell Rep.

[21] Wang J, Karra R, Dickson AL, Poss KD (2013). Fibronectin is deposited by injury-activated epicardial cells and is necessary for zebrafish heart regeneration. Dev Biol.

[22] Kikuchi K, Holdway JE, Major RJ, Blum N, Dahn RD, Begemann G (2011). Retinoic acid production by endocardium and epicardium is an injury response essential for zebrafish heart regeneration. Dev Cell.

[23] Bednarek D, González-Rosa JM, Guzmán-Martínez G, Gutiérrez-Gutiérrez Ó, Aguado T, Sánchez-Ferrer C (2015). Telomerase is essential for zebrafish heart regeneration. Cell Rep.

[24] Gemberling M, Karra R, Dickson AL, Poss KD (2015). Nrg1 is an injury-induced cardiomyocyte mitogen for the endogenous heart regeneration program in zebrafish. Elife.

[25] Zhao L, Borikova AL, Ben-Yair R, Guner-Ataman B, Macrae CA, Lee RT (2014). Notch signaling regulates cardiomyocyte proliferation during zebrafish heart regeneration. Proc Natl Acad Sci USA.

[26] Pfefferli C, Jaźwińska A (2017). The careg element reveals a common regulation of regeneration in the zebrafish myocardium and fin. Nat Commun.

[27] Gupta V, Poss KD (2012). Clonally dominant cardiomyocytes direct heart morphogenesis. Nature.

[28] Cui M, Atmanli A, Morales MG, Tan W, Chen K, Xiao X (2021). Nrf1 promotes heart regeneration and repair by regulating proteostasis and redox balance. Nat Commun.

[29] Kachanova O, Lobov A, Malashicheva A (2022). The role of the Notch signaling pathway in recovery of cardiac function after myocardial infarction. Int J Mol Sci.

[30] Ma J, Gu Y, Liu J, Song J, Zhou T, Jiang M (2022). Functional screening of congenital heart disease risk loci identifies 5 genes essential for heart development in zebrafish. Cell Mol Life Sci.

[31] Heallen T, Morikawa Y, Leach J, Tao G, Willerson JT, Johnson RL (2013). Hippo signaling impedes adult heart regeneration. Development.

[32] Leach JP, Heallen T, Zhang M, Rahmani M, Morikawa Y, Hill MC (2017). Hippo pathway deficiency reverses systolic heart failure after infarction. Nature.

[33] Aharonov A, Shakked A, Umansky KB, Savidor A, Genzelinakh A, Kain D (2020). ERBB2 drives YAP activation and EMT-like processes during cardiac regeneration. Nat Cell Biol.

[34] Fernández-Ruiz I (2021). ERBB2-YAP crosstalk mediates cardiac regeneration in mice. Nat Rev Cardiol.

[35] Xin M, Kim Y, Sutherland LB, Murakami M, Qi X, Mcanally J (2013). Hippo pathway effector Yap promotes cardiac regeneration. Proc Natl Acad Sci USA.

[36] Lin Z, von Gise A, Zhou P, Gu F, Ma Q, Jiang J (2014). Cardiac-specific YAP activation improves cardiac function and survival in an experimental murine MI model. Circ Res.

[37] Eulalio A, Mano M, Dal Ferro M, Zentilin L, Sinagra G, Zacchigna S (2012). Functional screening identifies miRNAs inducing cardiac regeneration. Nature.

[38] Lesizza P, Prosdocimo G, Martinelli V, Sinagra G, Zacchigna S, Giacca M (2017). Single-dose intracardiac injection of pro-regenerative microRNAs improves cardiac function after myocardial infarction. Circ Res.

[39] Gabisonia K, Prosdocimo G, Aquaro GD, Carlucci L, Zentilin L, Secco I (2019). MicroRNA therapy stimulates uncontrolled cardiac repair after myocardial infarction in pigs. Nature.

[40] Tao Y, Zhang H, Huang S, Pei L, Feng M, Zhao X (2019). miR-199a-3p promotes cardiomyocyte proliferation by inhibiting Cd151 expression. Biochem Biophys Res Commun.

[41] Li Z, Song Y, Liu L, Hou N, An X, Zhan D (2017). miR-199a impairs autophagy and induces cardiac hypertrophy through mTOR activation. Cell Death Differ.

[42] Hashemi Gheinani A, Burkhard FC, Rehrauer H, Aquino Fournier C, Monastyrskaya K (2015). microRNA miR-199a-5p regulates smooth muscle cell proliferation and morphology by targeting WNT2 signaling pathway. J Biol Chem.

[43] Huang W, Feng Y, Liang J, Yu H, Wang C, Wang B (2018). Loss of microRNA-128 promotes cardiomyocyte proliferation and heart regeneration. Nat Commun.

[44] Porrello ER, Mahmoud AI, Simpson E, Johnson BA, Grinsfelder D, Canseco D (2013). Regulation of neonatal and adult mammalian heart regeneration by the miR-15 family. Proc Natl Acad Sci USA.

[45] Valussi M, Besser J, Wystub-Lis K, Zukunft S, Richter M, Kubin T*,* et al*.* Repression of Osmr and Fgfr1 by miR-1/133a prevents cardiomyocyte dedifferentiation and cell cycle entry in the adult heart. Sci Adv. 2021;7(42):eabi6648. 10.1126/sciadv.abi6648 PMC8514096 34644107

[46] Huang S, Li X, Zheng H, Si X, Li B, Wei G (2019). Loss of super-enhancer-regulated circRNA Nfix induces cardiac regeneration after myocardial infarction in adult mice. Circulation.

[47] Wang X, Ha T, Liu L, Hu Y, Kao R, Kalbfleisch J (2018). TLR3 mediates repair and regeneration of damaged neonatal heart through glycolysis dependent YAP1 regulated miR-152 expression. Cell Death Differ.

[48] Yang YS, Liu MH, Yan ZW, Chen GQ, Huang Y. FAM122A is required for mesendodermal and cardiac differentiation of embryonic stem cells. Stem cells. 2023;sxad008. 10.1093/stmcls/sxad008. 10.1093/stmcls/sxad008 PMC10498146 36715298

[49] Rigaud VOC, Hoy RC, Kurian J, Zarka C, Behanan M, Brosious I (2023). RNA-binding protein LIN28a regulates new myocyte formation in the heart through long noncoding RNA-H19. Circulation.

[50] Ye Z, Su Z, Xie S, Liu Y, Wang Y, Xu X (2020). Yap-lin28a axis targets let7-Wnt pathway to restore progenitors for initiating regeneration. Elife.

[51] Gamba L, Amin-Javaheri A, Kim J, Warburton D, Lien CL (2017). Collagenolytic activity is associated with scar resolution in zebrafish hearts after cryoinjury. J Cardiovasc Dev Dis.

[52] Beauchemin M, Smith A, Yin VP (2015). Dynamic microRNA-101a and Fosab expression controls zebrafish heart regeneration. Development.

[53] Flinn MA, Jeffery BE, O'Meara CC, Link BA (2019). Yap is required for scar formation but not myocyte proliferation during heart regeneration in zebrafish. Cardiovasc Res.

[54] Simões FC, Cahill TJ, Kenyon A, Gavriouchkina D, Vieira JM, Sun X (2020). Macrophages directly contribute collagen to scar formation during zebrafish heart regeneration and mouse heart repair. Nat Commun.

[55] Chablais F, Veit J, Rainer G, Jaźwińska A (2011). The zebrafish heart regenerates after cryoinjury-induced myocardial infarction. BMC Dev Biol.

[56] González-Rosa JM, Martín V, Peralta M, Torres M, Mercader N (2011). Extensive scar formation and regression during heart regeneration after cryoinjury in zebrafish. Development.

[57] Bersell K, Arab S, Haring B, Kühn B (2009). Neuregulin1/ErbB4 signaling induces cardiomyocyte proliferation and repair of heart injury. Cell.

[58] D'Uva G, Aharonov A, Lauriola M, Kain D, Yahalom-Ronen Y, Carvalho S (2015). ERBB2 triggers mammalian heart regeneration by promoting cardiomyocyte dedifferentiation and proliferation. Nat Cell Biol.

[59] de Preux Charles AS, Bise T, Baier F, Marro J, Jaźwińska A. Distinct effects of inflammation on preconditioning and regeneration of the adult zebrafish heart. Open Biol. 2016;6(7):160102. 10.1098/rsob.160102 PMC4967830 27440424

[60] Fang Y, Gupta V, Karra R, Holdway JE, Kikuchi K, Poss KD (2013). Translational profiling of cardiomyocytes identifies an early Jak1/Stat3 injury response required for zebrafish heart regeneration. Proc Natl Acad Sci USA.

[61] Wen X, Jiao L, Tan H (2022). MAPK/ERK pathway as a central regulator in vertebrate organ regeneration. Int J Mol Sci.

[62] Zhang D, Ning J, Ramprasath T, Yu C, Zheng X, Song P (2022). Kynurenine promotes neonatal heart regeneration by stimulating cardiomyocyte proliferation and cardiac angiogenesis. Nat Commun.

[63] Missinato MA, Saydmohammed M, Zuppo DA, Rao KS, Opie GW, Kühn B*,* et al*.* Dusp6 attenuates Ras/MAPK signaling to limit zebrafish heart regeneration. Development. 2018;145(5):dev157206. 10.1242/dev.157206 PMC5868992 29444893

[64] Zhou X, Zhang C, Wu X, Hu X, Zhang Y, Wang X (2022). Dusp6 deficiency attenuates neutrophil-mediated cardiac damage in the acute inflammatory phase of myocardial infarction. Nat Commun.

[65] Zhang Z, Chen Y, Zheng L, Du J, Wei S, Zhu X*,* et al*.* A DUSP6 inhibitor suppresses inflammatory cardiac remodeling and improves heart function after myocardial infarction. Dis Model Mech. 2023;16(5):dmm049662. 10.1242/dmm.049662 PMC9789401 36478044

[66] Belviso I, Sacco AM, Cozzolino D, Nurzynska D, Di Meglio F, Castaldo C (2022). Cardiac-derived extracellular matrix: a decellularization protocol for heart regeneration. PLoS ONE.

[67] Han L, Mich-Basso JD, Li Y, Ammanamanchi N, Xu J, Bargaje AP (2022). Changes in nuclear pore numbers control nuclear import and stress response of mouse hearts. Dev Cell.

[68] Sogo T, Nakao S, Tsukamoto T, Ueyama T, Harada Y, Ihara D (2023). Canonical Wnt signaling activation by chimeric antigen receptors for efficient cardiac differentiation from mouse embryonic stem cells. Inflamm Regen.

[69] Senyo SE, Steinhauser ML, Pizzimenti CL, Yang VK, Cai L, Wang M (2013). Mammalian heart renewal by pre-existing cardiomyocytes. Nature.

[70] Kimura W, Xiao F, Canseco DC, Muralidhar S, Thet S, Zhang HM (2015). Hypoxia fate mapping identifies cycling cardiomyocytes in the adult heart. Nature.

[71] Porrello ER, Mahmoud AI, Simpson E, Hill JA, Richardson JA, Olson EN (2011). Transient regenerative potential of the neonatal mouse heart. Science.

[72] Orlic D, Kajstura J, Chimenti S, Jakoniuk I, Anderson SM, Li B (2001). Bone marrow cells regenerate infarcted myocardium. Nature.

[73] van Berlo JH, Kanisicak O, Maillet M, Vagnozzi RJ, Karch J, Lin SC (2014). C-kit^+^ cells minimally contribute cardiomyocytes to the heart. Nature.

[74] Li J, Yang KY, Tam RCY, Chan VW, Lan HY, Hori S (2019). Regulatory T-cells regulate neonatal heart regeneration by potentiating cardiomyocyte proliferation in a paracrine manner. Theranostics.

[75] Li J, Liang C, Yang KY, Huang X, Han MY, Li X (2020). Specific ablation of CD4^+^ T-cells promotes heart regeneration in juvenile mice. Theranostics.

[76] Das S, Goldstone AB, Wang H, Farry J, D'Amato G, Paulsen MJ (2019). A unique collateral artery development program promotes neonatal heart regeneration. Cell.

[77] Marín-Juez R, El-Sammak H, Helker CSM, Kamezaki A, Mullapuli ST, Bibli SI (2019). Coronary revascularization during heart regeneration is regulated by epicardial and endocardial cues and forms a scaffold for cardiomyocyte repopulation. Dev Cell.

[78] Marín-Juez R, Marass M, Gauvrit S, Rossi A, Lai SL, Materna SC (2016). Fast revascularization of the injured area is essential to support zebrafish heart regeneration. Proc Natl Acad Sci USA.

[79] Wang L, Yu P, Zhou B, Song J, Li Z, Zhang M (2020). Single-cell reconstruction of the adult human heart during heart failure and recovery reveals the cellular landscape underlying cardiac function. Nat Cell Biol.

[80] Jonsson MKB, Hartman RJG, Ackers-Johnson M, Tan WLW, Lim B, Van Veen TaB*,* et al*.* A transcriptomic and epigenomic comparison of fetal and adult human cardiac fibroblasts reveals novel key transcription factors in adult cardiac fibroblasts. JACC Basic Transl Sci. 2016;1(7):590–602. 10.1016/j.jacbts.2016.07.007 PMC6113540 30167544

[81] Kaur H, Takefuji M, Ngai CY, Carvalho J, Bayer J, Wietelmann A (2016). Targeted ablation of periostin-expressing activated fibroblasts prevents adverse cardiac remodeling in mice. Circ Res.

[82] Sánchez-Iranzo H, Galardi-Castilla M, Sanz-Morejón A, González-Rosa JM, Costa R, Ernst A (2018). Transient fibrosis resolves via fibroblast inactivation in the regenerating zebrafish heart. Proc Natl Acad Sci USA.

[83] Yuan P, Cheedipudi SM, Rouhi L, Fan S, Simon L, Zhao Z (2021). Single-cell RNA sequencing uncovers paracrine functions of the epicardial-derived cells in arrhythmogenic cardiomyopathy. Circulation.

[84] Xia H, Li X, Gao W, Fu X, Fang RH, Zhang L (2018). Tissue repair and regeneration with endogenous stem cells. Nat Rev Mater.

[85] Bergmann O, Zdunek S, Felker A, Salehpour M, Alkass K, Bernard S (2015). Dynamics of cell generation and turnover in the human heart. Cell.

[86] Haubner BJ, Schneider J, Schweigmann U, Schuetz T, Dichtl W, Velik-Salchner C (2016). Functional recovery of a human neonatal heart after severe myocardial infarction. Circ Res.

[87] Cesna S, Eicken A, Juenger H, Hess J (2013). Successful treatment of a newborn with acute myocardial infarction on the first day of life. Pediatr Cardiol.

[88] Deutsch MA, Cleuziou J, Noebauer C, Eicken A, Vogt M, Hoerer J (2014). Successful management of neonatal myocardial infarction with ECMO and intracoronary r-tPA lysis. Congenit Heart Dis.

[89] Farooqi KM, Sutton N, Weinstein S, Menegus M, Spindola-Franco H, Pass RH (2012). Neonatal myocardial infarction: case report and review of the literature. Congenit Heart Dis.

[90] Drenckhahn JD, Schwarz QP, Gray S, Laskowski A, Kiriazis H, Ming Z (2008). Compensatory growth of healthy cardiac cells in the presence of diseased cells restores tissue homeostasis during heart development. Dev Cell.

[91] Darehzereshki A, Rubin N, Gamba L, Kim J, Fraser J, Huang Y (2015). Differential regenerative capacity of neonatal mouse hearts after cryoinjury. Dev Biol.

[92] Haubner BJ, Adamowicz-Brice M, Khadayate S, Tiefenthaler V, Metzler B, Aitman T (2012). Complete cardiac regeneration in a mouse model of myocardial infarction. Aging.

[93] Bryant DM, O'Meara CC, Ho NN, Gannon J, Cai L, Lee RT (2015). A systematic analysis of neonatal mouse heart regeneration after apical resection. J Mol Cell Cardiol.

[94] Konfino T, Landa N, Ben-Mordechai T, Leor J (2015). The type of injury dictates the mode of repair in neonatal and adult heart. J Am Heart Assoc.

[95] González-Rosa JM, Burns CE, Burns CG (2017). Zebrafish heart regeneration: 15 years of discoveries. Regeneration (Oxf).

[96] Wang J, Panáková D, Kikuchi K, Holdway JE, Gemberling M, Burris JS (2011). The regenerative capacity of zebrafish reverses cardiac failure caused by genetic cardiomyocyte depletion. Development.

[97] Schultheiss HP, Fairweather D, Caforio ALP, Escher F, Hershberger RE, Lipshultz SE (2019). Dilated cardiomyopathy. Nat Rev Dis Primers.

[98] Cao J, Poss KD (2018). The epicardium as a hub for heart regeneration. Nat Rev Cardiol.

[99] Schächinger V, Erbs S, Elsässer A, Haberbosch W, Hambrecht R, Hölschermann H (2006). Intracoronary bone marrow-derived progenitor cells in acute myocardial infarction. N Engl J Med.

[100] Perin EC, Willerson JT, Pepine CJ, Henry TD, Ellis SG, Zhao DXM (2012). Effect of transendocardial delivery of autologous bone marrow mononuclear cells on functional capacity, left ventricular function, and perfusion in chronic heart failure: the FOCUS-CCTRN trial. JAMA.

[101] Sürder D, Manka R, Lo Cicero V, Moccetti T, Rufibach K, Soncin S (2013). Intracoronary injection of bone marrow-derived mononuclear cells early or late after acute myocardial infarction: effects on global left ventricular function. Circulation.

[102] Traverse JH, Henry TD, Pepine CJ, Willerson JT, Zhao DX, Ellis SG (2012). Effect of the use and timing of bone marrow mononuclear cell delivery on left ventricular function after acute myocardial infarction: the TIME randomized trial. JAMA.

[103] Menasché P, Alfieri O, Janssens S, Mckenna W, Reichenspurner H, Trinquart L (2008). The Myoblast Autologous Grafting in Ischemic Cardiomyopathy (MAGIC) trial: first randomized placebo-controlled study of myoblast transplantation. Circulation.

[104] Fouts K, Fernandes B, Mal N, Liu J, Laurita KR (2006). Electrophysiological consequence of skeletal myoblast transplantation in normal and infarcted canine myocardium. Heart Rhythm.

[105] Dominguez MH, Krup AL, Muncie JM, Bruneau BG (2023). Graded mesoderm assembly governs cell fate and morphogenesis of the early mammalian heart. Cell.

[106] Mummery C, Ward-Van Oostwaard D, Doevendans P, Spijker R, Van Den Brink S, Hassink R (2003). Differentiation of human embryonic stem cells to cardiomyocytes: role of coculture with visceral endoderm-like cells. Circulation.

[107] Mummery CL, Zhang J, Ng ES, Elliott DA, Elefanty AG, Kamp TJ (2012). Differentiation of human embryonic stem cells and induced pluripotent stem cells to cardiomyocytes: a methods overview. Circ Res.

[108] Shiba Y, Fernandes S, Zhu WZ, Filice D, Muskheli V, Kim J (2012). Human ES-cell-derived cardiomyocytes electrically couple and suppress arrhythmias in injured hearts. Nature.

[109] Chong JJH, Yang X, Don CW, Minami E, Liu YW, Weyers JJ (2014). Human embryonic-stem-cell-derived cardiomyocytes regenerate non-human primate hearts. Nature.

[110] Romagnuolo R, Masoudpour H, Porta-Sánchez A, Qiang B, Barry J, Laskary A (2019). Human embryonic stem cell-derived cardiomyocytes regenerate the infarcted pig heart but induce ventricular tachyarrhythmias. Stem Cell Rep.

[111] Menasché P, Vanneaux V, Hagège A, Bel A, Cholley B, Parouchev A (2018). Transplantation of human embryonic stem cell-derived cardiovascular progenitors for severe ischemic left ventricular dysfunction. J Am Coll Cardiol.

[112] Shiba Y, Gomibuchi T, Seto T, Wada Y, Ichimura H, Tanaka Y (2016). Allogeneic transplantation of iPS cell-derived cardiomyocytes regenerates primate hearts. Nature.

[113] Kawamura M, Miyagawa S, Miki K, Saito A, Fukushima S, Higuchi T (2012). Feasibility, safety, and therapeutic efficacy of human induced pluripotent stem cell-derived cardiomyocyte sheets in a porcine ischemic cardiomyopathy model. Circulation.

[114] Hare JM, Fishman JE, Gerstenblith G, Difede Velazquez DL, Zambrano JP, Suncion VY (2012). Comparison of allogeneic vs autologous bone marrow-derived mesenchymal stem cells delivered by transendocardial injection in patients with ischemic cardiomyopathy: the POSEIDON randomized trial. JAMA.

[115] Mathiasen AB, Qayyum AA, Jørgensen E, Helqvist S, Fischer-Nielsen A, Kofoed KF (2015). Bone marrow-derived mesenchymal stromal cell treatment in patients with severe ischaemic heart failure: a randomized placebo-controlled trial (MSC-HF trial). Eur Heart J.

[116] Bartolucci J, Verdugo FJ, González PL, Larrea RE, Abarzua E, Goset C (2017). Safety and efficacy of the intravenous infusion of umbilical cord mesenchymal stem cells in patients with heart failure: a phase 1/2 randomized controlled trial (RIMECARD Trial [Randomized Clinical Trial of Intravenous Infusion Umbilical Cord Mesenchymal Stem Cells on Cardiopathy]). Circ Res.

[117] Bartunek J, Terzic A, Davison BA, Filippatos GS, Radovanovic S, Beleslin B (2017). Cardiopoietic cell therapy for advanced ischaemic heart failure: results at 39 weeks of the prospective, randomized, double blind, sham-controlled CHART-1 clinical trial. Eur Heart J.

[118] Bartunek J, Terzic A, Davison BA, Behfar A, Sanz-Ruiz R, Wojakowski W (2020). Cardiopoietic stem cell therapy in ischaemic heart failure: long-term clinical outcomes. ESC Heart Fail.

[119] Yamada S, Bartunek J, Behfar A, Terzic A (2021). Mass customized outlook for regenerative heart failure care. Int J Mol Sci.

[120] Bolli R, Mitrani RD, Hare JM, Pepine CJ, Perin EC, Willerson JT (2021). A phase II study of autologous mesenchymal stromal cells and c-kit positive cardiac cells, alone or in combination, in patients with ischaemic heart failure: the CCTRN CONCERT-HF trial. Eur J Heart Fail.

[121] Bolli R, Hare JM, March KL, Pepine CJ, Willerson JT, Perin EC (2018). Rationale and design of the CONCERT-HF trial (combination of mesenchymal and c-kit^+^ cardiac stem cells as regenerative therapy for heart failure). Circ Res.

[122] Gao LR, Pei XT, Ding QA, Chen Y, Zhang NK, Chen HY (2013). A critical challenge: dosage-related efficacy and acute complication intracoronary injection of autologous bone marrow mesenchymal stem cells in acute myocardial infarction. Int J Cardiol.

[123] Ward MR, Abadeh A, Connelly KA (2018). Concise review: rational use of mesenchymal stem cells in the treatment of ischemic heart disease. Stem Cells Transl Med.

[124] Antonitsis P, Ioannidou-Papagiannaki E, Kaidoglou A, Papakonstantinou C (2007). In vitro cardiomyogenic differentiation of adult human bone marrow mesenchymal stem cells. The role of 5-azacytidine. Interact Cardiovasc Thorac Surg.

[125] Silva GV, Litovsky S, Assad JAR, Sousa ALS, Martin BJ, Vela D (2005). Mesenchymal stem cells differentiate into an endothelial phenotype, enhance vascular density, and improve heart function in a canine chronic ischemia model. Circulation.

[126] Xu JY, Qian HY, Huang PS, Xu J, Xiong YY, Jiang WY (2019). Transplantation efficacy of autologous bone marrow mesenchymal stem cells combined with atorvastatin for acute myocardial infarction (TEAM-AMI): rationale and design of a randomized, double-blind, placebo-controlled, multi-center Phase II TEAM-AMI trial. Regen Med.

[127] Vagnozzi RJ, Maillet M, Sargent MA, Khalil H, Johansen AKZ, Schwanekamp JA (2020). An acute immune response underlies the benefit of cardiac stem cell therapy. Nature.

[128] Bolli R, Tang XL, Guo Y, Li Q (2021). After the storm: an objective appraisal of the efficacy of c-kit+ cardiac progenitor cells in preclinical models of heart disease. Can J Physiol Pharmacol.

[129] Bolli R, Solankhi M, Tang XL, Kahlon A (2022). Cell therapy in patients with heart failure: a comprehensive review and emerging concepts. Cardiovasc Res.

[130] Nakajima H, Ishikawa H, Yamamoto T, Chiba A, Fukui H, Sako K (2023). Endoderm-derived islet1-expressing cells differentiate into endothelial cells to function as the vascular HSPC niche in zebrafish. Dev Cell.

[131] Akbarzadeh A, Sobhani S, Soltani Khaboushan A, Kajbafzadeh AM (2023). Whole-heart tissue engineering and cardiac patches: challenges and promises. Bioengineering (Basel).

[132] Diaz-Navarro R, Urrútia G, Cleland JG, Poloni D, Villagran F, Acosta-Dighero R*,* et al*.* Stem cell therapy for dilated cardiomyopathy. Cochrane Database Syst Rev. 2021;7(7):CD013433. 10.1002/14651858.CD013433.pub2 PMC8406792 34286511

[133] Weiss JN, Weiss JN (2022). IMMNC-HF: IntraMyocardial injection of bone marrow monoNuclear cells in heart failure (HF) patients. Stem cell surgery trials in heart failure and diabetes: a concise guide.

[134] Borow KM, Yaroshinsky A, Greenberg B, Perin EC (2019). Phase 3 DREAM-HF trial of mesenchymal precursor cells in chronic heart failure. Circ Res.

[135] Yamada S, Jeon R, Garmany A, Behfar A, Terzic A (2021). Screening for regenerative therapy responders in heart failure. Biomark Med.

[136] Tripathi H, Domingues A, Donahue R, Cras A, Guerin CL, Gao E (2023). Combined transplantation of human MSCs and ECFCs improves cardiac function and decrease cardiomyocyte apoptosis after acute myocardial infarction. Stem Cell Rev Rep.

[137] Spiroski AM, McCracken IR, Thomson A, Magalhaes-Pinto M, Lalwani MK, Newton KJ (2022). Human embryonic stem cell-derived endothelial cell product injection attenuates cardiac remodeling in myocardial infarction. Front Cardiovasc Med.

[138] Rolland L, Harrington A, Faucherre A, Abaroa JM, Gangatharan G, Gamba L*,* et al*.* The regenerative response of cardiac interstitial cells. J Mol Cell Biol. 2022. mjac059. 10.1093/jmcb/mjac059. 10.1093/jmcb/mjac059 PMC10068904 36271843

[139] Mohamed TMA, Ang YS, Radzinsky E, Zhou P, Huang Y, Elfenbein A (2018). Regulation of cell cycle to stimulate adult cardiomyocyte proliferation and cardiac regeneration. Cell.

[140] Liu S, Li K, Wagner Florencio L, Tang L, Heallen TR, Leach JP*,* et al*.* Gene therapy knockdown of Hippo signaling induces cardiomyocyte renewal in pigs after myocardial infarction. Sci Transl Med. 2021;13(600):eabd6892. 10.1126/scitranslmed.abd6892 PMC9476348 34193613

[141] Dang CV (2013). MYC, metabolism, cell growth, and tumorigenesis. Cold Spring Harb Perspect Med.

[142] Bywater MJ, Burkhart DL, Straube J, Sabò A, Pendino V, Hudson JE (2020). Reactivation of Myc transcription in the mouse heart unlocks its proliferative capacity. Nat Commun.

[143] Chen Y, Lüttmann FF, Schoger E, Schöler HR, Zelarayán LC, Kim KP (2021). Reversible reprogramming of cardiomyocytes to a fetal state drives heart regeneration in mice. Science.

[144] Polizzotti BD, Ganapathy B, Walsh S, Choudhury S, Ammanamanchi N, Bennett DG*,* et al*.* Neuregulin stimulation of cardiomyocyte regeneration in mice and human myocardium reveals a therapeutic window. Sci Transl Med. 2015;7(281):281ra45. 10.1126/scitranslmed.aaa5171 PMC5360874 25834111

[145] Lenihan DJ, Anderson SA, Lenneman CG, Brittain E, Muldowney JaS, 3rd, Mendes L*,* et al*.* A phase I, single ascending dose study of cimaglermin Alfa (neuregulin 1β3) in patients with systolic dysfunction and heart failure. JACC Basic Transl Sci. 2016;1(7):576–86. 10.1016/j.jacbts.2016.09.005 PMC6113538 30167542

[146] Gao R, Zhang J, Cheng L, Wu X, Dong W, Yang X (2010). A phase II, randomized, double-blind, multicenter, based on standard therapy, placebo-controlled study of the efficacy and safety of recombinant human neuregulin-1 in patients with chronic heart failure. J Am Coll Cardiol.

[147] Nguyen NUN, Canseco DC, Xiao F, Nakada Y, Li S, Lam NT (2020). A calcineurin-Hoxb13 axis regulates growth mode of mammalian cardiomyocytes. Nature.

[148] Mahmoud AI, Kocabas F, Muralidhar SA, Kimura W, Koura AS, Thet S (2013). Meis1 regulates postnatal cardiomyocyte cell cycle arrest. Nature.

[149] Pinto AR, Ilinykh A, Ivey MJ, Kuwabara JT, D'antoni ML, Debuque R (2016). Revisiting cardiac cellular composition. Circ Res.

[150] Ieda M, Fu JD, Delgado-Olguin P, Vedantham V, Hayashi Y, Bruneau BG (2010). Direct reprogramming of fibroblasts into functional cardiomyocytes by defined factors. Cell.

[151] Song K, Nam YJ, Luo X, Qi X, Tan W, Huang GN (2012). Heart repair by reprogramming non-myocytes with cardiac transcription factors. Nature.

[152] Abad M, Hashimoto H, Zhou H, Morales MG, Chen B, Bassel-Duby R (2017). Notch inhibition enhances cardiac reprogramming by increasing MEF2C transcriptional activity. Stem Cell Rep.

[153] Zhou H, Morales MG, Hashimoto H, Dickson ME, Song K, Ye W (2017). ZNF281 enhances cardiac reprogramming by modulating cardiac and inflammatory gene expression. Genes Dev.

[154] Yamakawa H, Muraoka N, Miyamoto K, Sadahiro T, Isomi M, Haginiwa S (2015). Fibroblast growth factors and vascular endothelial growth factor promote cardiac reprogramming under defined conditions. Stem Cell Rep.

[155] Zhou H, Dickson ME, Kim MS, Bassel-Duby R, Olson EN (2015). Akt1/protein kinase B enhances transcriptional reprogramming of fibroblasts to functional cardiomyocytes. Proc Natl Acad Sci USA.

[156] Zhou Y, Wang L, Vaseghi HR, Liu Z, Lu R, Alimohamadi S (2016). Bmi1 is a key epigenetic barrier to direct cardiac reprogramming. Cell Stem Cell.

[157] Mohamed TMA, Stone NR, Berry EC, Radzinsky E, Huang Y, Pratt K (2017). Chemical enhancement of in vitro and in vivo direct cardiac reprogramming. Circulation.

[158] Wang L, Ma H, Huang P, Xie Y, Near D, Wang H*,* et al*.* Down-regulation of Beclin1 promotes direct cardiac reprogramming. Sci Transl Med. 2020;12(566):eaay7856. 10.1126/scitranslmed.aay7856 PMC8188650 33087505

[159] Jayawardena TM, Egemnazarov B, Finch EA, Zhang L, Payne JA, Pandya K (2012). MicroRNA-mediated in vitro and in vivo direct reprogramming of cardiac fibroblasts to cardiomyocytes. Circ Res.

[160] Jayawardena TM, Finch EA, Zhang L, Zhang H, Hodgkinson CP, Pratt RE (2015). MicroRNA induced cardiac reprogramming in vivo: evidence for mature cardiac myocytes and improved cardiac function. Circ Res.

[161] Lalit PA, Salick MR, Nelson DO, Squirrell JM, Shafer CM, Patel NG (2016). Lineage reprogramming of fibroblasts into proliferative induced cardiac progenitor cells by defined factors. Cell Stem Cell.

[162] Tang Y, Aryal S, Geng X, Zhou X, Fast VG, Zhang J (2022). TBX20 improves contractility and mitochondrial function during direct human cardiac reprogramming. Circulation.

[163] Nam YJ, Song K, Luo X, Daniel E, Lambeth K, West K (2013). Reprogramming of human fibroblasts toward a cardiac fate. Proc Natl Acad Sci USA.

[164] Paoletti C, Divieto C, Tarricone G, Di Meglio F, Nurzynska D, Chiono V (2020). MicroRNA-mediated direct reprogramming of human adult fibroblasts toward cardiac phenotype. Front Bioeng Biotechnol.

[165] Wada R, Muraoka N, Inagawa K, Yamakawa H, Miyamoto K, Sadahiro T (2013). Induction of human cardiomyocyte-like cells from fibroblasts by defined factors. Proc Natl Acad Sci USA.

[166] Fu JD, Stone NR, Liu L, Spencer CI, Qian L, Hayashi Y (2013). Direct reprogramming of human fibroblasts toward a cardiomyocyte-like state. Stem Cell Rep.

[167] Islas JF, Liu Y, Weng KC, Robertson MJ, Zhang S, Prejusa A (2012). Transcription factors ETS2 and MESP1 transdifferentiate human dermal fibroblasts into cardiac progenitors. Proc Natl Acad Sci USA.

[168] Nam YJ, Lubczyk C, Bhakta M, Zang T, Fernandez-Perez A, Mcanally J (2014). Induction of diverse cardiac cell types by reprogramming fibroblasts with cardiac transcription factors. Development.

[169] Tani H, Sadahiro T, Yamada Y, Isomi M, Yamakawa H, Fujita R (2023). Direct reprogramming improves cardiac function and reverses fibrosis in chronic myocardial infarction. Circulation.

[170] Puente BN, Kimura W, Muralidhar SA, Moon J, Amatruda JF, Phelps KL (2014). The oxygen-rich postnatal environment induces cardiomyocyte cell-cycle arrest through DNA damage response. Cell.

[171] Nakada Y, Canseco DC, Thet S, Abdisalaam S, Asaithamby A, Santos CX (2017). Hypoxia induces heart regeneration in adult mice. Nature.

[172] Harada K, Friedman M, Lopez JJ, Wang SY, Li J, Prasad PV (1996). Vascular endothelial growth factor administration in chronic myocardial ischemia. Am J Physiol.

[173] House SL, Bolte C, Zhou M, Doetschman T, Klevitsky R, Newman G (2003). Cardiac-specific overexpression of fibroblast growth factor-2 protects against myocardial dysfunction and infarction in a murine model of low-flow ischemia. Circulation.

[174] Gyöngyösi M, Khorsand A, Zamini S, Sperker W, Strehblow C, Kastrup J (2005). NOGA-guided analysis of regional myocardial perfusion abnormalities treated with intramyocardial injections of plasmid encoding vascular endothelial growth factor A-165 in patients with chronic myocardial ischemia: subanalysis of the EUROINJECT-ONE multicenter double-blind randomized study. Circulation.

[175] Simons M, Annex BH, Laham RJ, Kleiman N, Henry T, Dauerman H (2002). Pharmacological treatment of coronary artery disease with recombinant fibroblast growth factor-2: double-blind, randomized, controlled clinical trial. Circulation.

[176] Zangi L, Lui KO, Von Gise A, Ma Q, Ebina W, Ptaszek LM (2013). Modified mRNA directs the fate of heart progenitor cells and induces vascular regeneration after myocardial infarction. Nat Biotechnol.

[177] Boos F, Oo JA, Warwick T, Günther S, Izquierdo Ponce J, Lopez M (2023). The endothelial-enriched lncRNA LINC00607 mediates angiogenic function. Basic Res Cardiol.

[178] Anttila V, Saraste A, Knuuti J, Hedman M, Jaakkola P, Laugwitz KL (2023). Direct intramyocardial injection of VEGF mRNA in patients undergoing coronary artery bypass grafting. Mol Ther.

[179] Arora H, Lavin AC, Balkan W, Hare JM, White IA (2021). Neuregulin-1, in a conducive milieu with Wnt/BMP/retinoic acid, prolongs the epicardial-mediated cardiac regeneration capacity of neonatal heart explants. J Stem Cells Regen Med.

[180] Wasserman AH, Huang AR, Lewis-Israeli YR, Dooley MD, Mitchell AL, Venkatesan M (2022). Oxytocin promotes epicardial cell activation and heart regeneration after cardiac injury. Front Cell Dev Biol.

[181] Smart N, Risebro CA, Melville AAD, Moses K, Schwartz RJ, Chien KR (2007). Thymosin beta4 induces adult epicardial progenitor mobilization and neovascularization. Nature.

[182] Smart N, Bollini S, Dubé KN, Vieira JM, Zhou B, Davidson S (2011). De novo cardiomyocytes from within the activated adult heart after injury. Nature.

[183] Barile L, Lionetti V, Cervio E, Matteucci M, Gherghiceanu M, Popescu LM (2014). Extracellular vesicles from human cardiac progenitor cells inhibit cardiomyocyte apoptosis and improve cardiac function after myocardial infarction. Cardiovasc Res.

[184] Gladka MM, Johansen AKZ, van Kampen SJ, Peters MMC, Molenaar B, Versteeg D*,* et al*.* Thymosin β4 and prothymosin α promote cardiac regeneration post-ischemic injury in mice. Cardiovasc Res. 2022. cvac155. 10.1093/cvr/cvac155. 10.1093/cvr/cvac155 PMC10153422 36125329

[185] Colombo M, Raposo G, Théry C (2014). Biogenesis, secretion, and intercellular interactions of exosomes and other extracellular vesicles. Annu Rev Cell Dev Biol.

[186] Gao L, Wang L, Wei Y, Krishnamurthy P, Walcott GP, Menasché P*,* et al*.* Exosomes secreted by hiPSC-derived cardiac cells improve recovery from myocardial infarction in swine. Sci Transl Med. 2020;12(561):eaay1318. 10.1126/scitranslmed.aay1318. 10.1126/scitranslmed.aay1318 32938792

[187] Saha P, Sharma S, Korutla L, Datla SR, Shoja-Taheri F, Mishra R (2019). Biogenesis, secretion, and intercellular interactions of exosomes and other extracellular vesicles. Sci Transl Med.

[188] Cai CL, Martin JC, Sun Y, Cui L, Wang L, Ouyang K (2008). A myocardial lineage derives from Tbx18 epicardial cells. Nature.

[189] Zhou B, Ma Q, Rajagopal S, Wu SM, Domian I, Rivera-Feliciano J (2008). Epicardial progenitors contribute to the cardiomyocyte lineage in the developing heart. Nature.

[190] Zhou B, Honor LB, He H, Ma Q, Oh JH, Butterfield C (2011). Adult mouse epicardium modulates myocardial injury by secreting paracrine factors. J Clin Invest.

[191] Arrell DK, Rosenow CS, Yamada S, Behfar A, Terzic A (2020). Cardiopoietic stem cell therapy restores infarction-altered cardiac proteome. NPJ Regen Med.

[192] de Abreu RC, Fernandes H, da Costa Martins PA, Sahoo S, Emanueli C, Ferreira L (2020). Native and bioengineered extracellular vesicles for cardiovascular therapeutics. Nat Rev Cardiol.

[193] Lim GB (2020). An acellular artificial cardiac patch for myocardial repair. Nat Rev Cardiol.

[194] Chow A, Stuckey DJ, Kidher E, Rocco M, Jabbour RJ, Mansfield CA (2017). Human induced pluripotent stem cell-derived cardiomyocyte encapsulating bioactive hydrogels improve rat heart function post myocardial infarction. Stem Cell Rep.

[195] Shafei S, Khanmohammadi M, Ghanbari H, Nooshabadi VT, Tafti SHA, Rabbani S (2022). Effectiveness of exosome mediated miR-126 and miR-146a delivery on cardiac tissue regeneration. Cell Tissue Res.

[196] Sharma V, Manhas A, Gupta S, Dikshit M, Jagavelu K, Verma RS (2022). Fabrication, characterization and in vivo assessment of cardiogel loaded chitosan patch for myocardial regeneration. Int J Biol Macromol.

[197] Hu C, Liu W, Long L, Wang Z, Zhang W, He S (2022). Regeneration of infarcted hearts by myocardial infarction-responsive injectable hydrogels with combined anti-apoptosis, anti-inflammatory and pro-angiogenesis properties. Biomaterials.

[198] Grigorian-Shamagian L, Sanz-Ruiz R, Climent A, Badimon L, Barile L, Bolli R*,* et al*.* Insights into therapeutic products, preclinical research models, and clinical trials in cardiac regenerative and reparative medicine: where are we now and the way ahead. Current opinion paper of the ESC Working Group on Cardiovascular Regenerative and Reparative Medicine. Cardiovasc Res. 2021;117(6):1428–33. 10.1093/cvr/cvaa337 33258961

[199] Lovell-Badge R, Anthony E, Barker RA, Bubela T, Brivanlou AH, Carpenter M (2021). ISSCR guidelines for stem cell research and clinical translation: the 2021 update. Stem Cell Rep.

[200] Povsic TJ, Sanz-Ruiz R, Climent AM, Bolli R, Taylor DA, Gersh BJ (2021). Reparative cell therapy for the heart: critical internal appraisal of the field in response to recent controversies. ESC Heart Fail.

[201] Yamada S, Behfar A, Terzic A (2021). Regenerative medicine clinical readiness. Regen Med.

[202] Vermersch E, Jouve C, Hulot JS (2020). CRISPR/Cas9 gene-editing strategies in cardiovascular cells. Cardiovasc Res.

[203] Zhang Y, Karakikes I (2021). Translating genomic insights into cardiovascular medicine: opportunities and challenges of CRISPR-Cas9. Trends Cardiovasc Med.

[204] Musunuru K. CRISPR and cardiovascular diseases. Cardiovasc Res. 2022. cvac048. 10.1093/cvr/cvac048. 10.1093/cvr/cvac048 35388882

[205] Jiang L, Liang J, Huang W, Ma J, Park KH, Wu Z (2022). CRISPR activation of endogenous genes reprograms fibroblasts into cardiovascular progenitor cells for myocardial infarction therapy. Mol Ther.

[206] Theodoris CV, Zhou P, Liu L, Zhang Y, Nishino T, Huang Y*,* et al*.* Network-based screen in iPSC-derived cells reveals therapeutic candidate for heart valve disease. Science. 2021;371(6530):eabd0724. 10.1126/science.abd0724 PMC7880903 33303684

[207] Lin X, Liu Y, Liu S, Zhu X, Wu L, Zhu Y (2022). Nested epistasis enhancer networks for robust genome regulation. Science.

[208] Sendra M, de Dios Hourcade J, Temiño S, Sarabia AJ, Ocaña OH, Domínguez JN*,* et al*.* Cre recombinase microinjection for single-cell tracing and localised gene targeting. Development. 2023;150(3):dev201206. 10.1242/dev.201206. 10.1242/dev.201206 PMC10110498 36734327

[209] Assad H, Assad A, Kumar A (2023). Recent developments in 3D bio-printing and its biomedical applications. Pharmaceutics.

